# Pregnancy and birth complications and long‐term maternal mental health outcomes: A systematic review and meta‐analysis

**DOI:** 10.1111/1471-0528.17889

**Published:** 2024-06-18

**Authors:** Elizabeth O. Bodunde, Daire Buckley, Eimear O'Neill, Sukainah Al Khalaf, Gillian M. Maher, Karen O'Connor, Fergus P. McCarthy, Karolina Kublickiene, Karen Matvienko‐Sikar, Ali S. Khashan

**Affiliations:** ^1^ School of Public Health University College Cork Cork Ireland; ^2^ INFANT Research Centre University College Cork Cork Ireland; ^3^ Perinatal Mental Health Acute Mental Health Services (AMHS) and Child and Adolescent Mental Health Services (CAMHS), University College Cork Cork Ireland; ^4^ Mohammed Al‐Mana College for Medical Sciences Dammam Saudi Arabia; ^5^ RISE, Early Intervention in Psychosis Team South Lee Mental Health Services Cork Ireland; ^6^ Department of Psychiatry and Neurobehavioral Science University College Cork Cork Ireland; ^7^ Department of Obstetrics and Gynaecology Cork University Maternity Hospital Cork Ireland; ^8^ Division of Renal Medicine, Department of Clinical Science, Intervention and Technology Karolinska Institutet Stockholm Sweden

**Keywords:** anxiety disorders, birth, complications, depression, maternal mental health, postpartum, pregnancy, PTSD

## Abstract

**Background:**

Few studies have examined the associations between pregnancy and birth complications and long‐term (>12 months) maternal mental health outcomes.

**Objectives:**

To review the published literature on pregnancy and birth complications and long‐term maternal mental health outcomes.

**Search strategy:**

Systematic search of Cumulative Index to Nursing and Allied Health Literature (CINAHL), Excerpta Medica Database (Embase), PsycInfo®, PubMed® and Web of Science from inception until August 2022.

**Selection criteria:**

Three reviewers independently reviewed titles, abstracts and full texts.

**Data collection and analysis:**

Two reviewers independently extracted data and appraised study quality. Random‐effects meta‐analyses were used to calculate pooled estimates. The Meta‐analyses of Observational Studies in Epidemiology (MOOSE) guidelines were followed. The protocol was prospectively registered on the International Prospective Register of Systematic Reviews (PROSPERO: CRD42022359017).

**Main results:**

Of the 16 310 articles identified, 33 studies were included (3 973 631 participants). Termination of pregnancy was associated with depression (pooled adjusted odds ratio, aOR 1.49, 95% CI 1.20–1.83) and anxiety disorder (pooled aOR 1.43, 95% CI 1.20–1.71). Miscarriage was associated with depression (pooled aOR 1.97, 95% CI 1.38–2.82) and anxiety disorder (pooled aOR 1.24, 95% CI 1.11–1.39). Sensitivity analyses excluding early pregnancy loss and termination reported similar results. Preterm birth was associated with depression (pooled aOR 1.37, 95% CI 1.32–1.42), anxiety disorder (pooled aOR 0.97, 95% CI 0.41–2.27) and post‐traumatic stress disorder (PTSD) (pooled aOR 1.75, 95% CI 0.52–5.89). Caesarean section was not significantly associated with PTSD (pooled aOR 2.51, 95% CI 0.75–8.37). There were few studies on other mental disorders and therefore it was not possible to perform meta‐analyses.

**Conclusions:**

Exposure to complications during pregnancy and birth increases the odds of long‐term depression, anxiety disorder and PTSD.

## 1 INTRODUCTION

Pregnancy and birth complications are increasingly a major problem, in association with maternal comorbidities, maternal age, serious abnormalities in the baby, and medical conditions associated with labour and birth.^1^, ^2^ Although the majority of pregnancies are medically uneventful and result in a safe birth with no significant health concerns for the child, a range of pregnancy and birth complications occurs in up to 15%–40% of pregnancies globally.^3^, ^4^ These include peripartum complications, such as postpartum haemorrhage and pre‐eclampsia, and adverse pregnancy outcomes, such as fetal growth restriction, preterm birth and gestational diabetes mellitus.^5^, ^6^ There is evidence that these complications continue to affect maternal and child health after childbirth.^7^, ^8^

Several studies have explored mental and psychological presentations following pregnancy and birth complications.^9^, ^10^, ^11^, ^12^, ^13^ Maternal postpartum depression, anxiety disorder and stress have been the focus of these existing studies.^14^, ^15^, ^16^ For example, preterm birth and caesarean section (CS) have been shown to increase the risk for depression, anxiety disorder, dysphoria and stress in the immediate postpartum period.^17^, ^18^, ^19^ Other studies have reported that miscarriage increased the incidence of depressive episodes among women with bipolar disorder.^20^, ^21^ Bergink et al. demonstrated that having pre‐eclampsia and a somatic comorbidity resulted in a 32% increased risk of psychiatric illness.^22^ Although the morbidity of these mental disorders after pregnancy and birth is well known, knowledge about their impact beyond the first few months following birth is limited. Potential reasons for the development of mental disorders beyond the typical postpartum period are complex.^23^ They include a history of mental illness, intimate partner violence (IPV), increased physical discomfort from a medical intervention, physical life adjustments and difficult birth experiences.^23^, ^24^

Existing studies investigating adverse maternal mental health have yielded findings that mainly focused on the first 6 months following pregnancy and birth.^25^, ^26^, ^27^ Fewer studies have reported on long‐term maternal mental health outcomes after pregnancy and birth complications beyond the immediate postpartum period (i.e. from pregnancy to 12 months postpartum).^11^, ^13^, ^28^, ^29^, ^30^ The findings from these studies are inconsistent and the magnitude of risk differs across individual studies.^11^, ^13^, ^31^, ^32^, ^33^ The psychological impact of complications in pregnancy and birth is yet to be systematically examined and there is significantly less research on adverse long‐term maternal mental health outcomes. To our knowledge, there has not been a comprehensive meta‐analysis estimating rates of long‐term maternal mental health outcomes for women following pregnancy and birth complications.

Therefore, the aim of this systematic review was to synthesise the available evidence examining the association between pregnancy and childbirth complications and long‐term maternal mental health outcomes.

## 2 METHODS

### 2.1 Protocol registration and reporting

The protocol for this study was registered on PROSPERO, the international prospective register of systematic reviews, with registration number CRD42022359017, and subsequently published.^34^

### 2.2 Search methodology

A systematic literature search was conducted in the Cumulative Index to Nursing and Allied Health Literature (CINAHL), Excerpta Medica Database (Embase), PsycInfo®, PubMed® and Web of Science electronic databases from inception through August 2022 using a detailed search strategy. The systematic search was supplemented by searching the reference lists of eligible articles by hand. Search terms relating to complications of pregnancy, birth and adverse mental health disorders were combined according to the principles of Boolean logic. The full search strategy adapted in each of the databases is presented in Appendix S1. Studies were retrieved from the databases and organised and managed using the Rayyan web‐based management software (Rayyan Systems, Cambridge, MA, USA). Three reviewers (EOB, DB and EO'N) independently reviewed the titles and abstracts of all studies in duplicate, obtaining full texts when necessary. The full text of potentially eligible studies was screened independently in duplicate, and where consensus could not be reached a fourth reviewer was consulted (ASK).

### 2.3 Study inclusion and exclusion criteria

We followed the Meta‐analysis of Observational Studies in Epidemiology (MOOSE) reporting guidelines,^35^ described in Appendix S2. The population of interest included women who have had at least one pregnancy with any of the following pregnancy and birth complications as exposures of interest: pre‐eclampsia; pregnancy loss (miscarriage or termination of pregnancy); stillbirth; CS (elective and/or emergency); preterm birth (defined as birth <37 weeks of gestation); third‐ or fourth‐degree perineal laceration; neonatal intensive care unit (NICU) admission for >72 h; major obstetric haemorrhage; and birth injury/trauma. The comparator was no corresponding pregnancy and/or birth complication(s). The primary outcome was depression, anxiety disorder, post‐traumatic stress disorder (PTSD), substance use disorder, psychosis, schizophrenia or bipolar disorder diagnosed after the first 12 months postpartum. We included only observational studies in which a complication of pregnancy or birth was reported, and adverse maternal mental health outcomes after the first year following birth was the outcome of interest. For studies with different follow‐up periods, we considered the longest follow‐up period in the meta‐analysis. Only data from original peer‐reviewed studies were included. Complications of pregnancy and/or birth and maternal mental health could be measured using medical records, doctor‐diagnosed self‐reporting or validated questionnaires. We excluded case reports, editorials, conference abstracts and studies focused on women with a pre‐existing mental illness or with mental illness prior to 1 year postpartum.

### 2.4 Data extraction

Using a standardized data collection form, two reviewers (EOB and DB) independently extracted data from eligible studies. The information extracted included author, year of publication, study design, data source, definition of pregnancy or birth complications, assessment of outcome, sample size, variables adjusted for (if any), and crude and adjusted estimates. If not reported, we used raw data to calculate the crude odds ratios (ORs) and 95% confidence intervals (95% CIs). Where information was not directly available from the studies, authors were contacted to request this information. Only one of the five authors contacted for further information replied.

### 2.5 Risk of bias assessment

Three reviewers (EOB, SAK and GMM) independently performed quality assessment in duplicate, using the Newcastle–Ottawa Scale (NOS). The scale uses a ‘star system’ in which stars are assigned to show study quality based on the following three criteria: selection of the study groups; comparability of the groups; and ascertainment of the exposure and/or outcome of interest. The score ranged from 0 to 9. We considered scores of 0–3 stars as low quality, 4–6 stars as moderate quality and 7–9 stars as high quality. Any discrepancies on screening, extraction or quality assessment were resolved through discussion among the reviewers.

### 2.6 Statistical analysis

Data were analysed using ReviewManager (RevMan 5.4, www.cochrane.org). Random‐effects meta‐analyses were used to calculate the pooled OR of the associations between pregnancy and birth complications and long‐term adverse maternal mental health outcomes. The generic inverse method was used to include studies that do not report raw data. Studies were analysed separately if they provided both crude and adjusted estimates. Estimates from studies that accounted for potential confounding factors in the analysis phase were included as adjusted estimates. Forest plots were used to display overall (either crude or adjusted, with priority for adjusted estimates if both crude and adjusted estimates were reported) and adjusted estimates, with corresponding 95% CIs, where possible. Statistical heterogeneity was explored based on *I* ^2^ values: 0%–40% was considered low; 40%–75% was considered moderate; and >75% was considered high heterogeneity.^36^ We included only studies that reported an estimated measure of association (e.g. OR; hazard ratio, HR; relative risk, RR; or sufficient data to calculate an OR and 95% CI). The OR, HR and RR values were considered approximations of each other under the assumptions for rare diseases.^34^, ^37^ Where a meta‐analysis was not feasible, we performed a narrative synthesis using Popay's methodology.^35^ The following sensitivity analyses were decided a priori: study design (cohort vs case–control vs cross‐sectional), study quality (low vs moderate/high), measurement of outcome data (medical records vs self‐reported vs validated questionnaires) and length of follow‐up (12–60 months vs >60 months). We performed a post‐hoc sensitivity analysis to investigate the impact of the study definition of pregnancy loss and adverse mental health outcomes, and excluded studies that reported early pregnancy loss or termination.

## 3 RESULTS

**FIGURE 1:**
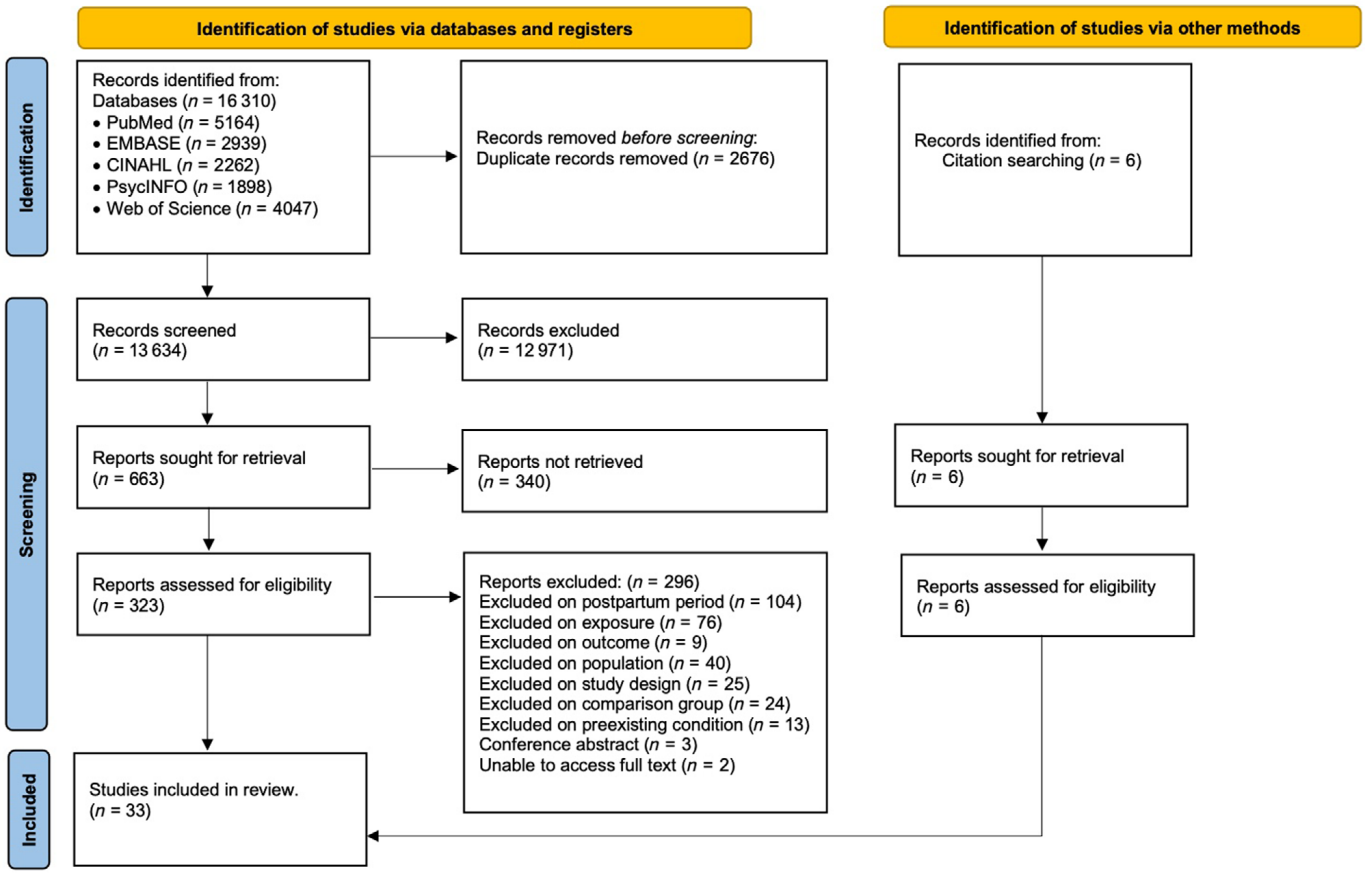
PRISMA flow diagram of studies selected for inclusion in this systematic review. Reproduced from Page et al.^79^

The study selection process is shown in the Preferred Reporting Items for Systematic reviews and Meta‐Analyses (PRISMA) flow diagram in Figure 1. In total, 16 310 articles were identified through database searching. An additional six articles were obtained through the manual searching of reference lists of relevant studies. After removing duplicates, the title and abstract of 13 634 articles were screened. The first evaluation of the title and abstract resulted in the exclusion of 12 971 articles. We further screened 663 articles, of which 323 articles were selected for full text review and 296 articles were excluded. Finally, 33 studies were eligible for inclusion in this review.

### 3.1 Characteristics of included studies

The 33 included studies were published from 1996 to 2022, with a total of 3 974 631 participants. The 33 studies were conducted in 15 countries: 14 studies were conducted in Europe^11^, ^13^, ^28^, ^31^, ^33^, ^38^, ^39^, ^40^, ^41^, ^42^, ^43^, ^44^, ^45^, ^46^; ten studies were conducted in North America^9^, ^29^, ^32^, ^47^, ^48^, ^49^, ^50^, ^51^, ^52^, ^53^; four studies were conducted in Asia^54^, ^55^, ^56^, ^57^; four studies were conducted in Australia^58^, ^59^, ^60^, ^61^; and one study was conducted in South America.^62^ Of these studies, 23 were prospective cohort studies^9^, ^11^, ^13^, ^28^, ^29^, ^32^, ^33^, ^39^, ^40^, ^41^, ^42^, ^43^, ^48^, ^49^, ^50^, ^51^, ^52^, ^53^, ^56^, ^58^, ^59^, ^60^, ^61^, ^62^; seven were retrospective cohort studies^43^, ^44^, ^45^, ^46^, ^47^, ^55^, ^57^; and three were case–control studies.^31^, ^38^, ^54^ The sample size ranged from 1 381 300 participants, in a population‐based study,^53^ to 46, in a single‐centre hospital‐based study.^13^ Studies reported mental health outcomes in relation to various pregnancy and birth complications: ten studies focused on preterm birth^9^, ^32^, ^39^, ^40^, ^43^, ^51^, ^52^, ^53^, ^58^, ^61^; seven studies focused on termination of pregnancy^11^, ^29^, ^38^, ^46^, ^49^, ^54^, ^62^; three studies focused on miscarriage,^33^, ^44^, ^59^ three studies focused on pre‐eclampsia^13^, ^28^, ^50^; three studies focused on stillbirth,^31^, ^42^, ^47^ three studies focused on CS^41^, ^45^, ^57^; and four studies reported results for both miscarriage and termination of pregnancy.^48^, ^55^, ^56^, ^60^ The most frequently reported mental health outcomes were depression and anxiety disorder (*n* = 29, 87.8%) and had an average length of follow‐up of 72 months. Twenty‐one (63.6%) of the included studies showed a moderate–high risk of bias and 12 studies (36.4%) had a low risk of bias. Table S1 contains a summary of the main findings from these studies.

### 3.2 Results of the meta‐analyses

The summary results of all the meta‐analyses that investigated the associations between pregnancy and birth complications and long‐term adverse mental health outcomes are presented in Table 1. A total of 21 studies provided 67 estimates of association measures and were included in the meta‐analyses.^9^, ^11^, ^28^, ^29^, ^42^, ^43^, ^44^, ^45^, ^46^, ^47^, ^48^, ^49^, ^50^, ^53^, ^55^, ^56^, ^57^, ^58^, ^59^, ^60^, ^61^

**TABLE 1:** Meta‐analyses of the association between pregnancy and birth complications and long‐term mental health outcomes.

Mental health outcomes	Pooled overall OR^a^		Pooled crude OR^b^				Pooled adjusted OR^c^			
	No. of studies	OR (95% CI)	No. of studies	No. of participants	OR (95% CI)	*I* ^2^ (%)	No. of studies	No. of participants	OR (95% CI)	*I* ^2^ (%)
Depression										
Termination of pregnancy	8	1.76 (1.36–2.28)	7	35 490	1.62 (1.11–2.36)	82	4	27 716	1.49 (1.20–1.86)	0
Miscarriage	7	2.09 (1.67–2.60)	6	37 274	2.12 (1.56–2.89)	70	4	29 623	1.97 (1.38–2.82)	52
Preterm birth	4	1.55 (1.15–2.09)	4	29 938	1.72 (1.17–2.52)	32	2	29 379	1.37 (1.32–1.42)	0
Pre‐eclampsia	2	1.16 (1.09–1.23)	2	1 210 963	1.12 (1.05–1.18)	0	2	1 210 963	1.16 (1.09–1.23)	0
Stillbirth	2	2.71 (2.29–3.21)	2	1 203 358	2.63 (2.23–3.10)	0	1	1 203 051	2.75 (2.31–3.27)	–
Caesarean section	1	1.32 (0.93–1.87)	–	–	–	–	1	25 238	1.32 (0.93–1.87)	–
Anxiety disorder										
Termination of pregnancy	6	1.44 (1.24–1.67)	5	10 046	1.65 (1.29–2.11)	29	4	27 963	1.43 (1.20–1.71)	16
Miscarriage	5	1.22 (1.11–1.34)	4	33 800	1.27 (1.05–1.53)	27	3	27 509	1.24 (1.11–1.39)	0
Preterm birth	3	1.44 (0.60–3.48)	3	45 818	1.57 (0.54–4.62)	78	2	45 688	0.97 (0.41–2.27)	87
Pre‐eclampsia	1	0.20 (0.05–0.87)	1	533	1.22 (0.84–1.77)	–	1	533	0.20 (0.05–0.87)	–
Stillbirth	2	2.30 (2.03–2.60)	2	1 203 358	2.74 (1.46–5.16)	46	1	1 203 051	2.32 (1.92–2.80)	–
Caesarean section	1	1.14 (0.95–1.37)	–	–	–	–	1	25 238	1.14 (0.95–1.37)	–
PTSD										
Termination of pregnancy	1	4.29 (1.93–9.55)	1	1295	4.29 (1.93–9.55)	–	–	–	–	–
Miscarriage	1	3.66 (1.78–7.53)	1	1360	3.66 (1.78–7.53)	–	–	–	–	–
Preterm birth	3	4.44 (2.79–7.09)	3	1929	5.08 (3.10–8.31)	0	1	155	1.75 (0.52–5.89)	–
Pre‐eclampsia	1	2.83 (1.91–4.19)	1	1531	2.83 (1.91–4.19)	–	–	–	–	–
Stillbirth	1	4.36 (2.31–8.23)	1	1 203 051	4.36 (2.31–8.23)	–	1	1 203 051	2.29 (1.95–2.68)	–
Third/fourth perineal tears	1	2.20 (1.38–3.51)	1	2376	2.20 (1.38–3.51)	–	–	–	–	–
Caesarean section	2	1.78 (1.34–2.36)	1	1182	7.71 (4.57–13.01)	–	2	26 420	2.51 (0.75–8.37)	91
Substance use disorder										
Termination of pregnancy	2	1.93 (1.29–2.88)	2	1980	2.34 (1.08–5.07)	77	1	936	2.30 (1.35–3.92)	–
Miscarriage	1	1.34 (0.70–2.56)	1	792	1.34 (0.70–2.56)	–	–	–	–	–
Stillbirth	1	2.41 (1.99–2.92)	1	1 203 051	2.56 (2.14–3.06)	–	1	1 203 051	2.41 (1.99–2.92)	–
Mood disorder										
Termination of pregnancy	1	1.18 (0.88–1.58)	1	936	1.89 (1.38–2.59)	–	1	936	1.18 (0.88–1.58)	–
Suicidal ideation										
Termination of pregnancy	1	1.25 (0.88–1.78)	1	936	3.37 (2.20–5.15)	–	1	936	1.25 (0.88–1.78)	–
Preterm birth	1	1.36 (1.28–1.44)	1	10 862	1.47 (1.39–1.55)	–	1	10 862	1.36 (1.28–1.44)	–
Stillbirth	1	3.16 (1.78–5.61)	1	1 203 051	3.09 (1.74–2.88)	–	1	1 203 051	3.16 (1.78–5.61)	–
Psychotic disorder										
Preterm birth	1	1.35 (1.25–1.46)	1	7163	1.39 (1.29–1.50)	–	1	7163	1.35 (1.25–1.46)	–
Stillbirth	1	2.27 (1.79–2.88)	1	1 203 051	2.29 (1.82–2.88)	–	1	1 203 051	2.27 (1.79–2.88)	–
Adjustment disorder										
Miscarriage	1	1.43 (1.23–2.25)	1	24 316	1.43 (1.23–2.25)	–	–	–	–	–
Stillbirth	1	4.15 (2.83–6.09)	1	1 203 051	5.18 (3.62–7.42)	–	1	1 203 051	4.15 (2.83–6.09)	–
Eating disorders										
Termination of pregnancy	1	1.82 (0.63–5.26)	1	936	2.54 (1.24–5.20)	–	1	936	1.82 (0.63–5.26)	–
Affective disorder										
Termination of pregnancy	1	14.82 (0.77–28.4)	1	89	14.82 (0.77–28.4)	–	–	–	–	–
Bipolar disorder										
Preterm birth	1	1.22 (1.15–1.29)	1	12 383	1.24 (1.17–1.31)	–	1	12 383	1.22 (1.15–1.29)	–
Personality disorder										
Preterm birth	1	1.50 (1.44–1.56)	1	19 434	1.59 (1053–1.65)	–	1	19 434	1.50 (1.44–1.56)	–

Abbreviations: CI, confidence interval; OR, odds ratio; PTSD, post‐traumatic stress disorder.

^a^ Overall pooled ORs presented a combination of adjusted (when reported by included studies) and crude (if adjusted estimates were not reported) estimates.

^b^ Only pooled estimate from studies reporting crude ORs.

^c^ Adjusted estimates following author's definition in individual studies; these studies adjusted for factors including maternal age, smoking, body mass index, pre‐existing mental health disorders, income, educational level, parity and other comorbidities.

### 3.3 Primary outcome: long‐term depression

A total of 16 studies reported estimates for depression.^9^, ^11^, ^28^, ^44^, ^46^, ^47^, ^48^, ^49^, ^50^, ^53^, ^55^, ^56^, ^58^, ^59^, ^60^, ^61^ For termination of pregnancy, seven unique studies reported crude estimates,^11^, ^46^, ^48^, ^49^, ^56^, ^59^, ^60^ and four studies reported adjusted estimates.^46^, ^49^, ^56^, ^59^ Figure S1 displays an overall pooled OR of 1.76 (95% CI 1.36–2.28), with a pooled OR of the adjusted estimates of 1.49 (95% CI 1.20–1.86) (*I* ^2^ = 0%) (Figure S2). For miscarriage, six studies reported crude estimates,^44^, ^46^, ^48^, ^55^, ^56^, ^59^ and four studies reported adjusted estimates,^44^, ^46^, ^55^, ^56^ with a pooled overall OR of 2.09 (95% CI 1.67–2.60) (Figure S1) and a pooled adjusted OR of 1.97 (95% CI 1.38–2.82) (*I* ^2^ = 52%). Moderate levels of heterogeneity were observed owing to one study,^44^ with the largest effect size. When this study was excluded, the pooled adjusted OR was 1.82 (95% CI 1.47–2.26) (*I* ^2^ = 0%) (Figure S2). For preterm birth, four studies reported crude estimates,^9^, ^53^, ^58^, ^61^ and two studies reported adjusted estimates,^53^, ^58^ with a pooled overall OR of 1.55 (95% CI 1.15–2.09) (Figure S1) and a pooled adjusted OR of 1.37 (95% CI 1.32–1.42) (*I* ^2^ = 0%) (Figure S2). Two studies reported on the association between pre‐eclampsia and depression,^28^, ^50^ producing on overall OR of 1.16 (95% CI 1.09–1.23), and the pooled adjusted OR was the same (Figure S1). Two studies reported estimates on depression among women who had experienced stillbirth,^42^, ^47^ with an overall OR of 2.71 (95% CI 2.29–3.21) (Figure S1).

### 3.4 Primary outcome: long‐term anxiety disorders

A total of 15 studies reported estimates for anxiety disorders.^9^, ^11^, ^28^, ^46^, ^47^, ^48^, ^49^, ^50^, ^53^, ^55^, ^56^, ^58^, ^59^, ^60^, ^61^ For the termination of pregnancy, five unique studies reported crude estimates,^11^, ^29^, ^55^, ^56^, ^59^ and four studies reported adjusted estimates.^29^, ^55^, ^56^, ^59^ Figure S3 displays a pooled overall OR of 1.44 (95% CI 1.24–1.67). The adjusted estimates remained almost unchanged, with a pooled adjusted OR of 1.43 (95% CI 1.20–1.71) (*I* ^2^ = 16%) (Figure S4). For miscarriage, four studies reported crude estimates,^46^, ^56^, ^59^, ^60^ and three studies reported adjusted estimates.^55^, ^56^, ^59^ Miscarriage was associated with a 1.2‐fold increase in the risk of anxiety disorder, with a pooled overall OR of 1.22 (95% CI 1.11–1.34) (Figure S3) and a pooled adjusted OR of 1.24 (95% CI 1.11–1.39) (*I* ^2^ = 0%) (Figure S4). For preterm birth, three studies reported crude estimates,^53^, ^58^, ^61^ and two studies reported adjusted estimates.^42^, ^47^ There was no association between preterm birth and the risk of anxiety disorder, with a pooled adjusted OR of 0.97 (95% CI 0.41–2.27), (*I* ^2^ = 87%) (Figure S4). The heterogeneity level remained high (*I* ^2^ = 76%) after we excluded one study with an outlying effect estimate.^61^ Two studies reported estimates on anxiety disorder among women who had experienced stillbirth, resulting in a pooled overall OR of 2.30 (95% CI 2.03–2.60) (*I* ^2^ = 0%) (Figure S3).^42^, ^47^ In addition, there was a suggestion that the odds of anxiety disorder in women who had pre‐eclampsia was reduced, with an adjusted OR of 0.20 (95% CI 0.05–0.87) (Figure S3). However, there was only one study on this association, which should be interpreted with caution.^28^

### 3.5 Other long‐term mental health outcomes

Findings from 12 unique studies were narratively reported as stand‐alone estimates, owing to the varying outcomes measures and summary scales used.^13^, ^31^, ^32^, ^33^, ^38^, ^39^, ^40^, ^41^, ^51^, ^52^, ^54^, ^62^ These studies were excluded from the meta‐analyses because the necessary data required were not reported. Table S2 contains a summary of the main findings from these studies. Five studies examined the association of preterm birth and adverse maternal mental health outcomes. Three of these studies found a significantly higher level of PTSD, anxiety disorder and depression in women who had experienced preterm birth.^39^, ^40^, ^52^ However, two studies reported no difference in the odds of depression and anxiety disorder among mothers of preterm infants.^32^, ^51^ Two of the three studies that examined the association between miscarriage and depression showed a higher risk of depression among women with miscarriage.^33^, ^62^ The study reported by Adib‐rad et al. suggested that at 18 months there was no association with somatisation among women who had a miscarriage, compared with the control group.^54^ One study found an association between pre‐eclampsia and PTSD.^13^ Two studies reported estimates of obsessive‐compulsive disorder (OCD),^33^, ^54^ but only one study reported a higher risk among women with miscarriage.^54^ Another study reported a combined outcome of depression and anxiety disorder as an outcome of interest and found that women were at greatest risk following emergency CS, compared with women who had a vaginal birth, at 3 years postpartum.^41^ A study reported that miscarriage (OR 2.16–2.36) and termination of pregnancy (OR 1.75–2.01) were both positively associated with the risk of psychiatric disorders; depression, anxiety disorder, adjustment disorder and somatoform disorder.^38^

### 3.6 Bias and heterogeneity

According to the NOS,^63^ the included studies scored between 2 and 8 stars, as shown in Table S3. Based on estimates in the meta‐analyses, the heterogeneity between studies was low for studies assessing outcomes of depression or anxiety disorder (*I* ^2^ = 0%–40%), and was high for studies assessing PTSD and substance use disorder (*I* ^2^ ≥75%). We were unable to perform a publication bias analysis because none of the meta‐analyses included ten or more estimates.^64^

### 3.7 Results of sensitivity analysis excluding early pregnancy loss and termination

#### 3.7.1 Long‐term depression

For termination of pregnancy, six studies reported crude estimates,^11^, ^46^, ^48^, ^49^, ^59^, ^60^ and three studies reported adjusted estimates,^29^, ^55^, ^59^ with a pooled crude OR (cOR) of 1.65 (95% CI 1.04–2.60) (*I* ^2^ = 84%) and a pooled aOR of 1.50 (95% CI 1.15–1.94) (*I* ^2^ = 0%). For miscarriage, four studies reported crude estimates,^46^, ^48^, ^59^, ^60^ and two studies reported adjusted estimates,^55^, ^59^ with a pooled cOR of 1.99 (95% CI 1.46–2.71) (*I* ^2^ = 66%) and a pooled aOR of 1.88 (95% CI 1.50–2.53), (*I* ^2^ = 0%) (Table 2).

**TABLE 2:** Summary results of the meta‐analyses excluding early pregnancy loss and termination of pregnancy.

Mental health outcomes	Pooled crude OR				Pooled adjusted OR			
	No. of studies	No. of participants	OR (95% CI)	*I* ^2^ (%)	No. of studies	No. of participants	OR (95% CI)	*I* ^2^ (%)
Depression								
Termination of pregnancy	6	28 603	1.65 (1.04–2.60)	84	3	20 829	1.50 (1.15–1.94)	0
Miscarriage	4	28 273	1.99 (1.46–2.71)	66	2	20 622	1.88 (1.50–2.35)	0
Anxiety disorder								
Termination of pregnancy	4	3159	1.56 (1.18–2.05)	31	3	21 076	1.37 (1.17–1.61)	0
Miscarriage	3	26 913	1.28 (1.07–1.54)	31	2	20 622	1.25 (1.11–1.40)	0
PTSD								
Termination of pregnancy	1	1295	4.29 (1.93–9.55)	–	–	–	–	–
Miscarriage	1	1360	3.66 (1.78–7.53)	–	–	–	–	–
Substance use disorder								
Termination of pregnancy	2	1980	2.34 (1.08–5.07)	77	1	936	2.30 (1.35–3.92)	–
Miscarriage	1	792	1.34 (0.70–2.56)	–	–	–	–	–
Mood disorder								
Termination of pregnancy	1	936	1.89 (1.38–2.59)	–	1	936	1.18 (0.88–1.58)	–
Suicidal ideation								
Termination of pregnancy	1	936	3.37 (2.20–5.15)	–	1	936	1.25 (0.88–1.78)	–
Adjustment disorder								
Miscarriage	1	24 316	1.43 (1.23–2.25)	–	–	–	–	–
Eating disorders								
Termination of pregnancy	1	936	2.54 (1.24–5.20)	–	1	936	1.82 (0.63–5.26)	–
Affective disorder								
Termination of pregnancy	1	89	14.82 (0.77–28.4)	–	–	–	–	–

*Note*: Only pooled estimates are listed from studies reporting crude odd ratios. Adjusted estimates follow the author's definition in individual studies; these studies adjusted for factors including maternal age, smoking, body mass index, pre‐existing mental health disorders, income, educational level, parity and other comorbidities.

Abbreviations: CI, confidence interval; OR, odds ratio; PTSD, post‐traumatic stress disorder.

#### 3.7.2 Long‐term anxiety disorders

For termination of pregnancy, four studies reported crude estimates,^29^, ^40^, ^59^, ^60^ and three studies reported adjusted estimates,^29^, ^55^, ^59^ with a pooled cOR of 1.56 (95% CI 1.18–2.05) (*I* ^2^ = 31%) and a pooled aOR of 1.37 (95% CI 1.17–1.61) (*I* ^2^ = 0%). For miscarriage, three studies reported crude estimates,^46^, ^59^, ^60^ and two studies reported adjusted estimates,^55^, ^59^ with a pooled cOR of 1.28 (95% CI 1.07–1.54) (*I* ^2^ = 31%) and pooled aOR of 1.25 (95% CI 1.11–1.40) (*I* ^2^ = 0%) (Table 2). Other mental disorders were not estimable because of the lack of studies, and their results remained the same (Table 2).

## 4 DISCUSSION

### 4.1 Main findings

The aim of this comprehensive systematic review and meta‐analysis was to examine the association between pregnancy and birth complications and long‐term adverse maternal health outcomes. Our findings suggest that termination of pregnancy, miscarriage, stillbirth, pre‐eclampsia and preterm birth were associated with an increase in the odds of long‐term depression, anxiety disorder and PTSD, compared with women who did not have complications in pregnancy and birth. This review, which includes findings up to August 2022, strengthens the previously suggested link between pregnancy and birth complications and adverse maternal mental health outcomes.

### 4.2 Interpretation

Previous studies have largely focused on the postpartum period and various postpartum psychiatric disorders^65^, ^66^; however, the knowledge available on the long‐term mental health outcomes is limited. In our meta‐analysis, the majority of included studies examined the associations between pregnancy loss and depression, anxiety disorder and PTSD. There was less research on preterm birth, pre‐eclampsia, CS and third‐ or fourth‐degree perineal tears. These findings highlight the likelihood that specific pregnancy and/or complications may be acute stressors with long‐term effects on the mental health of the mother. An unexpected and traumatic event such as pregnancy loss is significantly associated with the natural process of grieving, a high level of guilt and a loss of self.^67^, ^68^ Empirical literature suggests that non‐medical termination of pregnancy may not be traumatic for some women and/or in certain contexts, as a result of factors such as unplanned pregnancy and tokophobia.^23^, ^69^ However, it is possible that some women may subsequently experience a level of distress and experience trauma related to a non‐medical termination, which may contribute to long‐term mental health problems.^70^ In this review, we observed that the associations between pregnancy and birth complications such as preterm birth, stillbirth, pre‐eclampsia and long‐term PTSD were consistent; however, the findings for CS varied.^45^, ^57^ In this review, CS was examined regardless of whether it was an elective or emergency CS. Some studies report that higher levels of PTSD are expected in women who had unexpected birth outcomes in relation to elective CS.^21^, ^71^ The difference in findings could therefore be related to the type of CS.

This systematic review examined a range of mental health outcomes, with non‐psychotic mental disorders such as anxiety disorder, depression and PTSD as the most reported outcomes. Over 80% of all included studies focused on these outcomes, suggesting that these are considered highly important aspects of long‐term maternal mental health. This is unsurprising, in light of established research on maternal and perinatal mental health. Perinatal mental disorders other than non‐psychotic disorders have been scarcely researched and are given less consideration when examining maternal mental health.^72^ Outcomes such as psychosis, OCD, eating disorders and schizophrenia were reported less frequently or were absent in this review.

There is existing evidence suggesting that maternal mental disorders increase the risk of pregnancy and birth complications, substance use disorder and partner relationship difficulties.^73^, ^74^, ^75^, ^76^ It is therefore important to understand the trajectories of these mental health disorders and their impact on adverse pregnancy outcomes in subsequent pregnancies. The mechanisms underlying observed associations in this review are uncertain, and it is plausible that long‐term adverse mental health outcomes may relate to an underlying/unresolved predisposition caused by events in pregnancy and birth.^77^ One study suggested that termination of pregnancy for fetal anomaly or health concerns for pregnant women is a major life event that may cause sustained psychological morbidity.^78^

### 4.3 Strengths and limitations

This review has the following strengths. We followed a predefined, prospectively registered and peer‐reviewed protocol. We conducted a comprehensive search of five databases and supplemented this with hand‐searching the reference lists of the included studies. At least two independent reviewers carried out each process of the systematic review using a standardised data extraction form and validated study appraisal tool.

This review considered a wide range of complications in pregnancy and birth; however, this review did not cover all pregnancy and birth complications, and the findings should be interpreted with caution considering its limitations. We included termination of pregnancy, miscarriage, preterm labour and stillbirth in the same article to provide a comprehensive overview of the current state of the existing literature. Although these complications may have been studied individually in previous studies, to our knowledge, these associations have not been quantified in the form of a systematic review and meta‐analysis. The majority of studies lack any adjustment for pre‐existing mental health disorders during pregnancy. Although we excluded studies that included women with pre‐existing mental health disorders, some included studies did not explicitly exclude women with pre‐existing mental health issues or comorbidities.^9^, ^43^, ^48^, ^62^ The inclusion of these studies improves the statistical power of our meta‐analysis; however, they did not account for pre‐existing mental health disorders, thereby potentially skewing the results. With the small number of studies in each meta‐analysis, we were unable to conduct a sensitivity analysis to determine how including these studies impacted the overall results. Adjusted estimates were based on different sets of confounding factors across individual studies, from which only three studies accounted for pre‐existing mental health disorders.^28^, ^29^, ^60^ Most studies were of moderate–high risk of bias.^9^, ^11^, ^13^, ^28^, ^31^, ^32^, ^33^, ^39^, ^40^, ^41^, ^43^, ^44^, ^49^, ^51^, ^52^, ^54^, ^58^, ^59^, ^60^, ^61^, ^62^ Although 33 eligible studies were identified, the largest meta‐analysis included eight studies only. This was resolved by narratively summarising the results of the studies that were excluded from the meta‐analyses. Several diagnostic measures of mental health disorders were used. For example, some studies used International Classification of Diseases registry codes,^29^, ^46^, ^47^, ^50^, ^53^, ^57^ whereas other studies used questionnaires varying in reliability and validity.^28^, ^32^, ^56^, ^59^, ^60^, ^61^ Self‐report was the most frequently reported assessment method of mental health outcomes, which may potentially introduce reporting and misclassification bias if it was not based on validated tools.^9^, ^11^, ^13^, ^28^, ^29^, ^32^, ^33^, ^38^, ^40^, ^43^, ^46^, ^49^, ^51^, ^55^, ^56^, ^58^, ^59^, ^60^, ^61^ We were unable to conduct predefined subgroup meta‐analyses or assess publication bias owing to the small number of studies available. Two studies reported composite adverse mental health disorders without clear information relating to the specific diagnoses, which led to their exclusion from the meta‐analyses.^38^, ^41^ Most studies were conducted in Europe and North America, predominately among white populations; therefore, the results may not be generalisable to other populations. Overall, unaccounted factors such as maternal self‐efficacy, subsequent pregnancy complications, adverse life events, social support, child health problems and maternal comorbidities in the pooled estimates cannot be ruled out. Therefore, future population‐based studies should be designed to understand the role of potential confounding and mediation factors in these associations.

## 5 CONCLUSION

Our systematic review suggests that complications during pregnancy and birth increase the odds of long‐term depression, anxiety disorder and PTSD. However, because of the above limitations, a more definitive conclusion will require better designed studies on maternal mental health disorders. More data regarding maternal mental health outcomes beyond the typically examined postpartum period are needed to tailor the support and services for women with these mental health issues and to identify women more at risk of developing long‐term mental health disorders.

## AUTHOR CONTRIBUTIONS

Conception and design of the work: EOB, KO'C, FPM, KM‐S and ASK. Data search, extraction and checks: EOB, DB, EO'N, SAK and GMM. The evaluation and summary were drafted by all authors. All authors made significant contributions to drafting and/or revising the article. All authors approved the final version of the article for publication.

## FUNDING INFORMATION

This research was funded by the Health Research Board (SPHeRE‐2018‐1).

## CONFLICT OF INTEREST STATEMENT

The authors report no conflicts of interest.

## ETHICS APPROVAL

None required.

## Supporting information


Figure S1.

Figure S2.

Figure S3.

Figure S4.

Appendix S1.

## Data Availability

The data that support the findings of this study are available in the Supporting Information for this article.
