# Supplementary figures and images for: Pregnancy and birth complications and long‐term maternal mental health outcomes: A systematic review and meta‐analysis
Source: BJOG. 2024 Jun 18;132(2):131–42. doi: 10.1111/1471-0528.17889 (PMC11625657; doi:10.1111/1471-0528.17889)

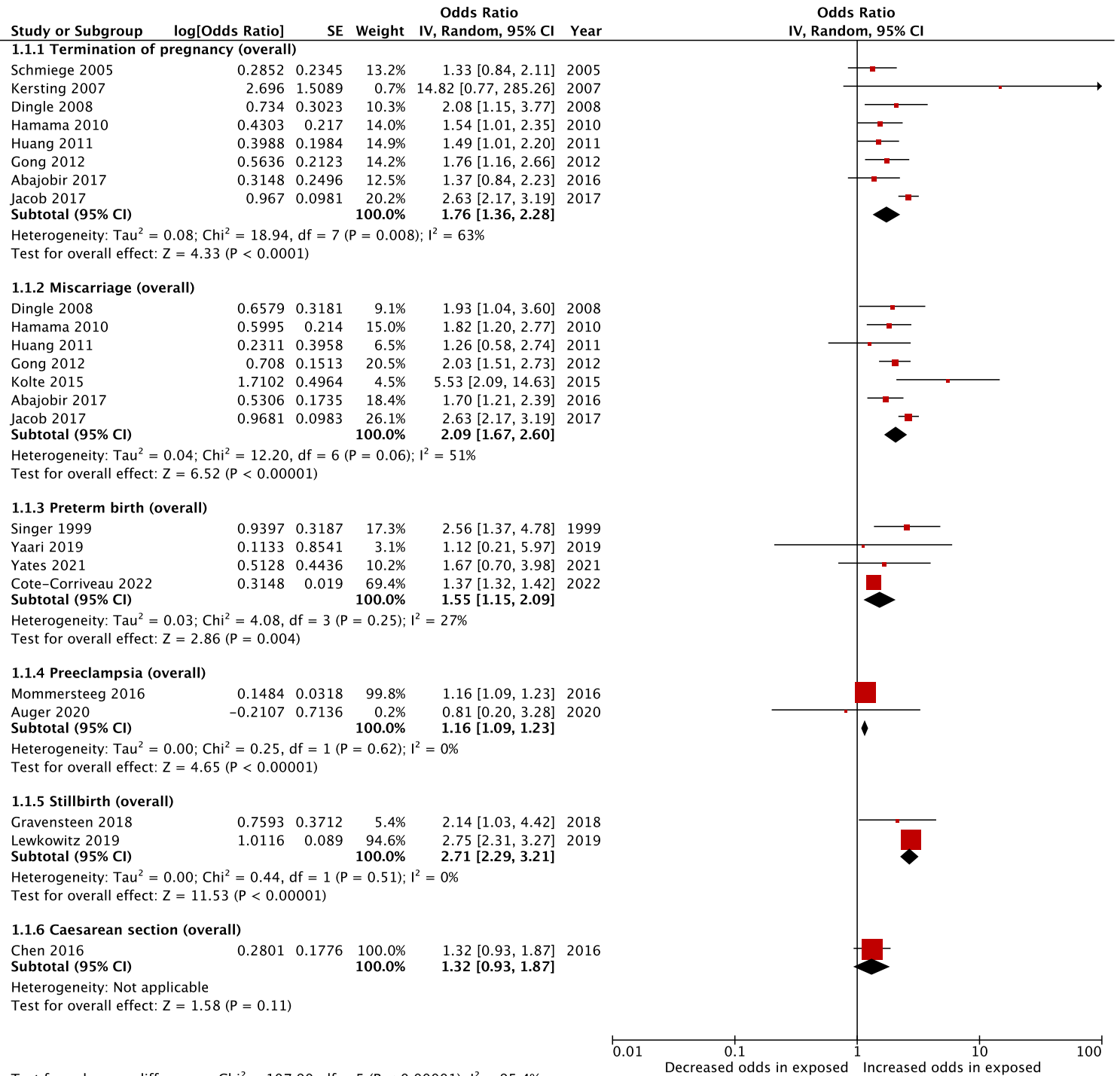

Supplement: Supplementary file 1 — Figure S1. [file BJO-132-131-s004.pdf]

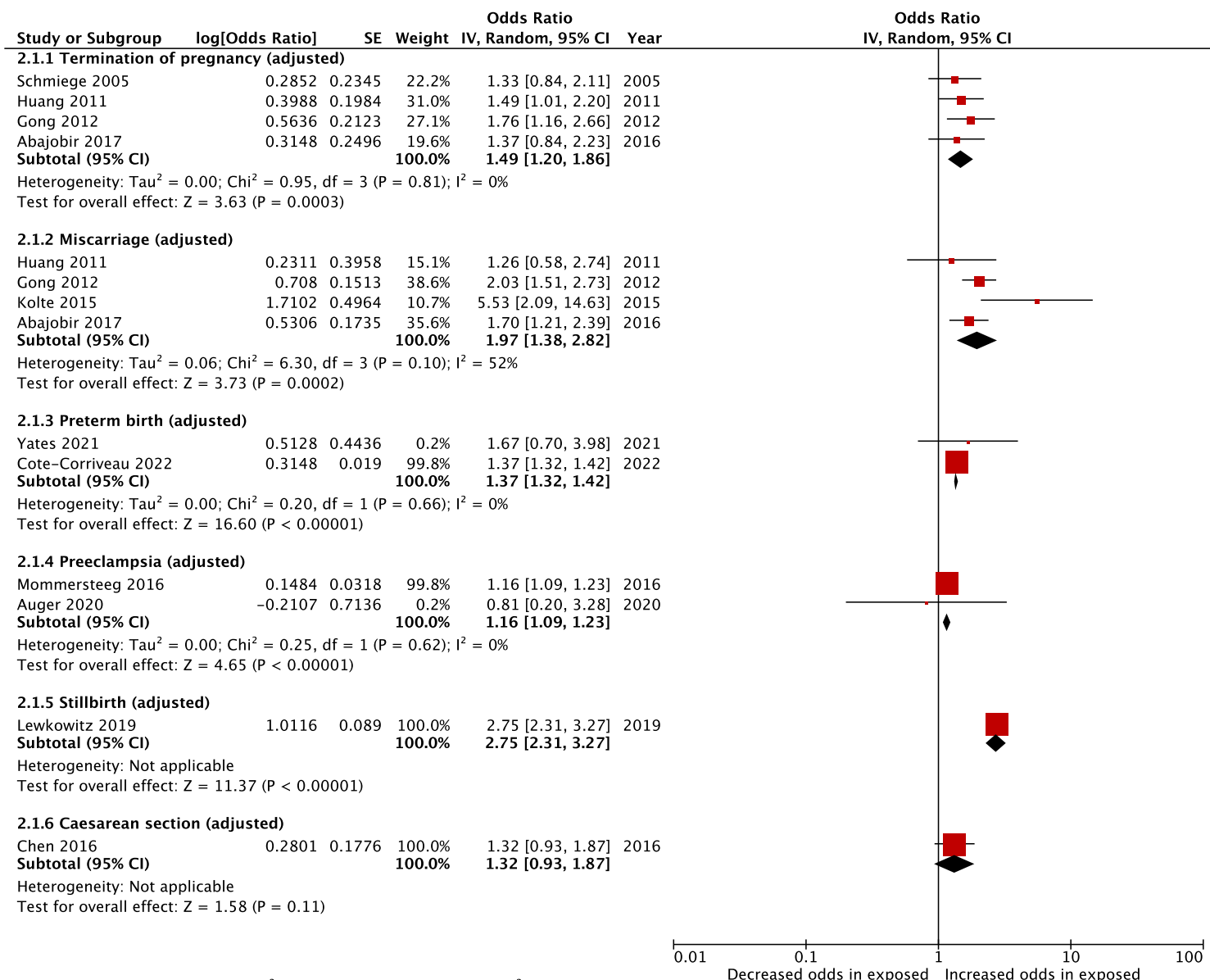

Supplement: Supplementary file 2 — Figure S2. [file BJO-132-131-s005.pdf]

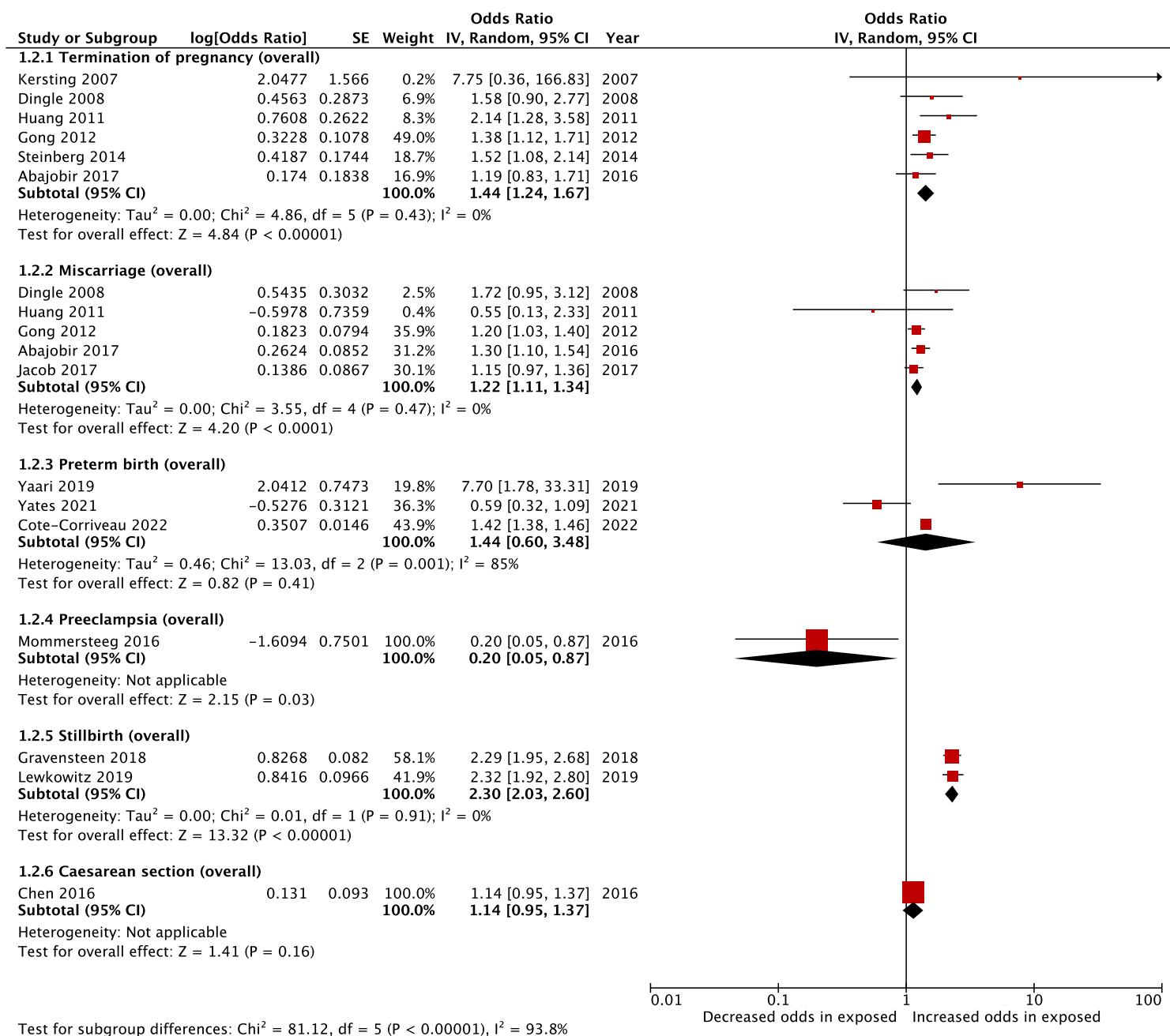

Supplement: Supplementary file 3 — Figure S3. [file BJO-132-131-s003.pdf]

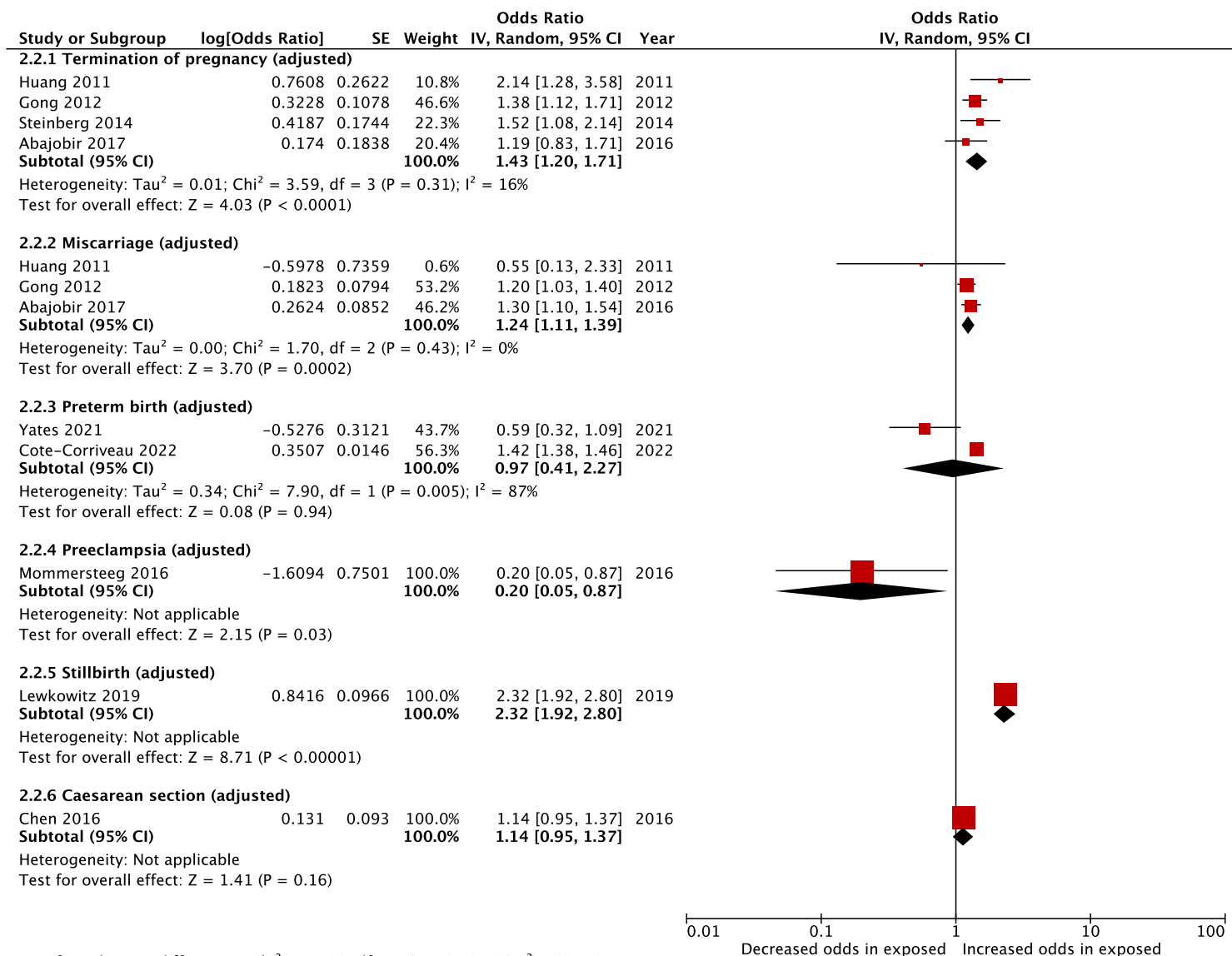

Test for subgroup differences:  $\chi^2 = 44.08$ ,  $df = 5$  ( $P < 0.00001$ ),  $I^2 = 88.7\%$

Supplement: Supplementary file 4 — Figure S4. [file BJO-132-131-s001.pdf]
