# Supplementary material for: Pregnancy and birth complications and long‐term maternal mental health outcomes: A systematic review and meta‐analysis
Source: BJOG. 2024 Jun 18;132(2):131–42. doi: 10.1111/1471-0528.17889 (PMC11625657; doi:10.1111/1471-0528.17889)
Supplement: Supplementary file 5 — Appendix S1. [file BJO-132-131-s002.docx]

**Supplementary Information**

Appendix S1: Search Strategy

Appendix S2: MOOSE Checklist

Table S1: Characteristics of included studies reporting the association between pregnancy and birth complications and adverse long-term maternal mental health outcomes.

Table S2: Characteristics of included studies reporting the association between pregnancy and birth complications and adverse long-term maternal mental health outcomes – Narrative reports.

Table S3: Newcastle-Ottawa studies quality assessment of included studies.

References.

**Appendix S1: Search strategy adapted for each electronic database.**

1. Pre-eclampsia
2. Preeclampsia
3. Pregnancy Toxemias
4. Pregnancy Toxemia
5. Toxemia, Pregnancy
6. Edema-Proteinuria-Hypertension Gestosis
7. Edema Proteinuria Hypertension Gestosis
8. Gestosis, Edema-Proteinuria-Hypertension
9. Hypertension-Edema-Proteinuria Gestosis
10. Gestosis, Hypertension-Edema-Proteinuria
11. Hypertension Edema Proteinuria Gestosis
12. Toxemia of Pregnancy
13. Haemorrhage, Early Pregnancy
14. Hemorrhage, Early Pregnancy
15. Severe Hemorrhage
16. Obstetric Hemorrhage
17. Obstetric Haemorrhage
18. Abortions, Spontaneous
19. Spontaneous Abortions
20. Spontaneous Abortion
21. Early Pregnancy Loss
22. Early Pregnancy Losses
23. Loss, Early Pregnancy
24. Losses, Early Pregnancy
25. Pregnancy Loss
26. Pregnancy Losses
27. Miscarriage
28. Miscarriages
29. Abortion, Tubal
30. Abortions, Tubal
31. Tubal Abortion
32. Tubal Abortions
33. Emergency Cesarean Section
34. Emergency Caesarean Section
35. Cesarean Sections
36. Delivery, Abdominal
37. Abdominal Deliveries
38. Caesarean Section
39. Caesarean Sections
40. Abdominal Delivery
41. C-Section (OB)
42. C Section (OB)
43. C-Sections (OB)
44. Postcesarean Section
45. Birth, Premature
46. Premature Births
47. Preterm Birth
48. Birth, Preterm
49. Births, Preterm
50. Neonatal intensive care unit admission
51. NICU admission
52. Intrauterine growth restriction
53. IUGR
54. Preterm Births
55. Perineal Tear
56. Perineal Trauma
57. Perineal Laceration
58. Perineum, Trauma
59. Perineum, Tear
60. Perineum, Laceration
61. Injuries, Birth
62. Birth Injury
63. Injury, Birth
64. Traumatic birth
65. #1 or #2 or #3 or #4 or #5 or #6 or #7 or #8 or #9 or #10 or #11 or #12 or #13 or #14 or #15 or #16 or #17 or #18 or #19 or #20 or #21 or #22 or #23 or #24 or #25 or #26 or #27 or #28 or #29 or #30 or #31 or #32 or #33 or #34 or #35 or #36 or #37 or #38 or #39 or #40 or #41 or #42 or #43 or #44 or #45 or #46 or #47 or #48 or #49 or #50 or #51 or #52 or #53 or #54 or #55 or #56 or #57 or #58 or #59 or #60 or #61 or #62 or #63 or #64
66. Maternal Mental Health
67. Anxiety Disorder
68. Disorder, Anxiety
69. Disorders, Anxiety
70. Neuroses, Anxiety
71. Anxiety Neuroses
72. Anxiety States, Neurotic
73. Anxiety State, Neurotic
74. Neurotic Anxiety State
75. Neurotic Anxiety States
76. State, Neurotic Anxiety
77. States, Neurotic Anxiety
78. Anxiety Symptoms
79. Anxiety Symptom
80. Symptoms, Anxiety
81. Depression
82. Depressive Symptoms
83. Depressive Symptom
84. Symptom, Depressive
85. Symptoms, Depressive
86. Emotional Depression
87. Depression, Emotional
88. Postpartum Depression
89. Postnatal Depression
90. Depression, Postnatal
91. Post-Partum Depression
92. Depression, Post-Partum
93. Post Partum Depression
94. Post-Natal Depression
95. Depression, Post-Natal
96. Post Natal Depression
97. Bipolar Disorders
98. Disorder, Bipolar
99. Affective Psychosis, Bipolar
100. Bipolar Affective Psychosis
101. Psychoses, Bipolar Affective
102. Psychosis, Bipolar Affective
103. Manic-Depressive Psychosis
104. Manic Depressive Psychosis
105. Psychosis, Manic-Depressive
106. Psychosis, Manic Depressive
107. Bipolar Mood Disorder
108. Bipolar Mood Disorders
109. Disorder, Bipolar Mood
110. Mood Disorder, Bipolar
111. Psychoses, Manic-Depressive
112. Psychoses, Manic Depressive
113. Depression, Bipolar
114. Bipolar Depression
115. Manic Depression
116. Depression, Manic
117. Depressions, Manic
118. Manic Disorder
119. Disorder, Manic
120. Manic Disorders
121. Schizophrenias
122. Schizophrenic Disorders
123. Disorder, Schizophrenic
124. Disorders, Schizophrenic
125. Schizophrenic Disorder
126. Disorder, Psychotic
127. Disorders, Psychotic
128. Psychotic Disorder
129. Psychosis
130. Psychoses
131. Schizoaffective Disorder
132. Disorder, Schizoaffective
133. Disorders, Schizoaffective
134. Schizoaffective Disorders
135. Schizophreniform Disorders
136. Disorder, Schizophreniform
137. Disorders, Schizophreniform
138. Schizophreniform Disorder
139. Psychosis, Brief Reactive
140. Brief Reactive Psychoses
141. Brief Reactive Psychosis
142. Psychoses, Brief Reactive
143. Reactive Psychoses, Brief
144. Reactive Psychosis, Brief
145. Disorder, Mood
146. Mood Disorder
147. Affective Disorders
148. Affective Disorder
149. Disorder, Affective
150. Chronic Post-Traumatic Stress Disorder
151. Chronic Post Traumatic Stress Disorder
152. Post-Traumatic Stress Disorder
153. Stress Disorder, Post-Traumatic
154. PTSD
155. Neuroses, Posttraumatic
156. Posttraumatic Neuroses
157. Post-Traumatic Stress Disorders
158. Stress Disorders, Posttraumatic
159. Post-Traumatic Stress Disorder
160. Delayed Onset Post-Traumatic Stress Disorder
161. Anorexia
162. Anorexia
163. Bulimia
164. Somatisation
165. Conversation disorder
166. Hypochondriasis
167. Body dysmorphic disorder
168. Adjustment disorders
169. #66 or #67 or #68 or #69 or #70 or #71 #72 or #73 or #74 or #75 or #76 or #77 or #78 or #79 or #80 or #81 #82 or #83 or #84 or #85 or #86 or #87 #88 or #89 or #90 or #91 or #92 or #93 or #94 or #95 or #96 or #97 or #98 or #99 or #100 or #101 or #102 or #103 or #104 or #105 or #106 or #107 or #108 or #109 or #110 or #111 or #112 or #113 or #114 or #115 or #116 or #117 #118 or #119 or #120 or #121 or #122 or #123 or #124 or #125 or #126 or #127 #128 or #129 or #130 or #131 or #132 or #133 or #134 or #135 or #136 or #137 or #138 or #139 or #140 or #141 or #142 or #143 or #144 or #145 or #146 or #147 or #148 or 149 or #150 or #151 or #152 or #153 or #154 or #155 or #156 or #157 or #158 or #159 or #160 or #161 or #162 or #163 or #164 or #165 or #166 or #167 or #168
170. #65 AND #169.

**Appendix S2: MOOSE Checklist for Meta-analyses of Observational Studies**

***Pregnancy and Birth Complications and Long-term Maternal Mental Health Outcomes: A Systematic Review and Meta-analysis.***

| **Item No** | **Recommendation** | **Reported on Page No** |
| --- | --- | --- |
| Reporting of background should include | |  |
| 1 | Problem definition | Manuscript Page 4 |
| 2 | Hypothesis statement | Manuscript Page 4 |
| 3 | Description of study outcome(s) | Manuscript Page 6 |
| 4 | Type of exposure or intervention used | Manuscript Page 6 |
| 5 | Type of study designs used | Manuscript Page 5-6 |
| 6 | Study population | Manuscript Page 6 |
| Reporting of search strategy should include | |  |
| 7 | Qualifications of searchers (eg, librarians and investigators) | Manuscript Page 5-6 |
| 8 | Search strategy, including time period included in the synthesis and key words | Manuscript Page 5-6 Appendix S1 |
| 9 | Effort to include all available studies, including contact with authors | Manuscript Page 7 |
| 10 | Databases and registries searched | Manuscript Page 5-6 |
| 11 | Search software used, name and version, including special features used (eg, explosion) | Manuscript Page 5 |
| 12 | Use of hand searching (eg, reference lists of obtained articles) | Manuscript Page 5 |
| 13 | List of citations located and those excluded, including justification | Manuscript Page 8-9 Figure 1 |
| 14 | Method of addressing articles published in languages other than English | - |
| 15 | Method of handling abstracts and unpublished studies | Manuscript Page 6  Figure 1 |
| 16 | Description of any contact with authors | Manuscript Page 7 |
| Reporting of methods should include | |  |
| 17 | Description of relevance or appropriateness of studies assembled for assessing the hypothesis to be tested | Manuscript Page 7-8  Table S1 –S2 |
| 18 | Rationale for the selection and coding of data (eg, sound clinical principles or convenience) | Manuscript p9-11 |
| 19 | Documentation of how data were classified and coded (eg, multiple raters, blinding and interrater reliability) | Manuscript Page 9  Table S1 –S2 |
| 20 | Assessment of confounding (eg, comparability of cases and controls in studies where appropriate) | Manuscript Page 7-8  Table S1 –S2 |
| 21 | Assessment of study quality, including blinding of quality assessors, stratification, or regression on possible predictors of study results | Manuscript Page 7-8, 12  Table S3 |
| 22 | Assessment of heterogeneity | Manuscript Page 10-11 |
| 23 | Description of statistical methods (eg, complete description of fixed or random effects models, justification of whether the chosen models account for predictors of study results, dose-response models, or cumulative meta-analysis) in sufficient detail to be replicated | Manuscript Page 7-8 |
| 24 | Provision of appropriate tables and graphics | Manuscript Table 1, Manuscript Table 2, Manuscript Figure 1-3B,  Table S1-S3 |
| Reporting of results should include | |  |
| 25 | Graphic summarizing individual study estimates and overall estimates | Manuscript Figure 2B, 2A, 3B, 3A |
| 26 | Table giving descriptive information for each study included | Table S1 –S3 |
| 27 | Results of sensitivity testing (eg, subgroup analysis) | Manuscript Page 12-13, Manuscript Table 2 |
| 28 | Indication of statistical uncertainty of findings | - |

| **Item No** | **Recommendation** | **Reported on Page No** |
| --- | --- | --- |
| Reporting of discussion should include | |  |
| 29 | Quantitative assessment of bias (e.g., publication bias) | - |
| 30 | Justification for exclusion (e.g., exclusion of non-English language citations) | Manuscript Page 6 |
| 31 | Assessment of quality of included studies | Manuscript Page 12  Table S3 |
| Reporting of conclusions should include | |  |
| 32 | Consideration of alternative explanations for observed results | Manuscript Page 12 -15 |
| 33 | Generalization of the conclusions (i.e., appropriate for the data presented and within the domain of the literature review) | Manuscript Page 12 -16 |
| 34 | Guidelines for future research | Manuscript Page 15-16 |
| 35 | Disclosure of funding source | Manuscript Page 17 |

*From*: Stroup DF, Berlin JA, Morton SC, et al. (2000) for the Meta-analysis Of Observational Studies in Epidemiology (MOOSE) Group. Meta-analysis of Observational Studies in Epidemiology. A Proposal for Reporting. JAMA 283(15):2008-2012. doi: 10.1001/jama.283.15.2008.

**Table S1: Characteristics of included studies reporting the association between pregnancy and birth complications and adverse long-term maternal mental health outcomes.**

| **Study, Year** | **Data Source** | **Country, study period** | **Study design,** | **Sample Size** | **Definition or assessment of pregnancy/ birth complication** | **Defined mental outcomes** | **Assessment method of Mental outcomes** | **Variables accounted for** | **Follow up period** |
| --- | --- | --- | --- | --- | --- | --- | --- | --- | --- |
| Steinberg J, 2014^1^ | National comorbidity survey replication data | United States  2001-2003 | Prospective cohort | 936 | Abortion defined according to the National Comorbidity Survey-Replication | Anxiety disorder, mood disorder, impulse-control substance use disorder, eating and suicidal ideation. | DSM-IV | Previous abortion, mental health problems, pregnancy adverse exposures, miscarriage, age at first pregnancy, childhood economic status. | 0-17 years |
| Kersting A, 2007^2^ | Medical records; University of Muenster. | Germany  2000-2003 | Prospective cohort | 127 | Abortion defined as induced termination of pregnancy in the 2nd or 3^rd^ trimester. | Complicated grief, PTSD, depression, anxiety, and psychiatric diagnoses. | SCID, DSM-IV. Perinatal Grief Scale, IES-R, BDI, State-Trait Anxiety inventory | Maternal age, marital status, education, religious faith stressful life events, gestational age, living children | 2 days - 14 months |
| Mommersteeg P, 2016^3^ | PREVFEM historical cohort study | The Netherland  2009-2013 | Prospective cohort | 533 | Preeclampsia defined as elevated diastolic blood pressure >90mmHg with proteinuria >0.3g/24h | Depressive symptoms, anxiety, and fatigue. | Depression -PHQ -9 Anxiety- GAD-7, Fatigue-FAS | Age, education, BMI, physical activity, presence of hypertension, marital status, personality scales, substance use, lifestyle and medical use. | 4 years |
| Lewkowitz A, 2019^4^ | Florida state inpatient database, and Agency for healthcare research and quality. | United  States  2005-2015 | Retrospective cohort | 1206046 | Stillbirth at >23 weeks’ gestation defined based on ICD – 9- CM codes. | Suicide depression, anxiety, psychosis, PTSD, acute stress reaction, adjustment disorder, substance use disorder. | ICD -9- CM codes | Age, race/ethnicity, payer, income, quartile zip code, mode of delivery, maternal medical comorbidities, and severe maternal morbidity. | >1 year |
| Abajobir A, 2017^5^ | MUSP | Australia  1981 - 1983 | Prospective cohort | 4403 | Miscarriage defined as previous history of miscarriage, still birth and ectopic pregnancy. Abortion defined as Termination of pregnancy*.* | Anxiety and depressive symptoms. | DSSI | Maternal age, education, marital status, annual family income, follow-up period. | 14 years |
| Chen H, 2017^6^ | Taiwan National Health Insurance database | Taiwan  1999 - 2008 | Retrospective cohort | 25238 | Cesarean section defined according to diagnosis related group. | Anxiety depression and stress symptoms | ICD-9- CM, | Residential urbanity, age, socioeconomic status, and antepartum comorbidity. | >1 year |
| Yaari M, 2019^7^ | Victorian Infant Brain Study | Australia  2001 - 2003 | Prospective cohort | 262 | Preterm birth defined as birth <30 weeks gestation or birth weight <1250g. | Anxiety, depression, social dysfunction, and somatic symptoms. | GHQ, HADS, | Familial social risk, child neonatal medical risk, child sex, multiple pregnancy, child’s neurodevelopmental impairment at 2 years. | 2- 13 years |
| Yates R, 2021^8^ | Victorian Infant Brain Study 2 | Australia  2011 - 2013 | Prospective cohort | 155 | Preterm birth is defined as <30 weeks gestation. NICU admission | Anxiety, depression, and PTSD | HADS, CES-D, and PCL-S. | Maternal age at birth, family structure, education level, employment status, income, first language. | 5 years |
| Huang Z, 2011^9^ | The ABCD cohort study | China  2008 - 2009 | Prospective cohort | 6887 | Induced and spontaneous abortion (defined by pregnancy loss at first and second trimester). | Depression and anxiety | SAS Zung’s Self-Rating Anxiety Scale, CES-D. | Maternal education, income, place of residence, and BMI. | >1 year |
| Jacob L, 2017^10^ | Disease analyzer database | Germany  2007 – 2015 | Retrospective cohort | 24316 | Spontaneous abortion defined according to ICD-10 O03. | Depression, anxiety, adjustment disorder | ICD -10 Codes | Age, index year, diagnosis of infertility, procreative management, physician. | >1 year |
| Schmiege S, 2005^11^ | US National longitudinal survey of youth | United states  1979 – 1992 | Prospective cohort | 689 | Abortion defined as unwanted first pregnancy | Depression | CES – Depression Scale | Race, age at first pregnancy, marital status, education, and family income | 13 years |
| Gong X, 2012^12^ | The C-ABC cohort study | China  2008 – 2010 | Retrospective cohort | 18204 | Miscarriage defined as fetal loss before 28 completed weeks of gestation, Abortion defined as abortion induced by artificial means. | Anxiety, Depression. | SAS, CES-D | Maternal age, maternal education, pre-pregnancy BMI, family income, place of residence. | 6 - >24 months |
| Hamama L, 2010^13^ | The STACY project | United states  2005-  2008 | Prospective cohort | 1581 | Elective and spontaneous abortion before 20 weeks of gestation | PTSD (within the past month), major depression | NWS-PTSD, CIDI | Obstetric history, race, age, income, education, crime rate in their zip code | >12 months |
| Suttora C, 2014^14^ | Online survey | Italy | Prospective cohort | 244 | Preterm birth defined as gestational age <37 weeks. | PTSD, Parenting stress, social support. | PPQ, PSI-SF, MSPSS | Not reported | 1-36 months |
| Dingle K, 2008^15^ | MUSP | Australia  2000 – 2004 | Prospective cohort | 1223 | Pregnancy loss defined as abortions and miscarriage (not specified). | Depression, anxiety, and substance use disorders | DSSI, PACS, CBCL | Early family life, history of mental disorders, adolescents’ behaviour, demographic factors. | 6 months -21 years |
| Kolte A, 2015^16^ | Online survey | Denmark  2010 - 2014 | Cross sectional | 2114 | Recurrent Pregnancy loss defined as > 3 consecutive pregnancy loss before 12 weeks gestation. | Depression and Emotional stress | MDI, Cohen PSS | Age, education, household income, number of live born children and number of pregnancies. | > 6  months (not specified) |
| Auger N, 2020^17^ | Hospital records / registry | Canada  1989 - 2016 | Prospective cohort | 1210963 | Preeclampsia defined according to 9th and 10th ICD Codes. | Depression defined as major depressive disorder requiring hospitalisation. | ICD Codes | Age at first delivery, total parity, substance use, comorbidity, pregnancy complication, socioeconomic disadvantage, time of the index pregnancy. | 0 - 28 years |
| Hernandez - Martinez A, 2019^18^ | Online survey | Spain | Cross -  sectional | 1531 | Cesarean section, Third / fourth tear, preeclampsia, and    NICU. | PTSD | PPQ | Mother age, parity, epidural use, mode of birth, sever tears, manual removal of placenta and skin-to-skin contact. | 1 - 5 years |
| Gravensteen I, 2018^19^ | Medical birth registry of Norway | Norway | Prospective cohort | 901 | Stillbirth defined as fetal death at 22 or more completed gestational weeks or birth weight >500g. | Anxiety, depression and relationship satisfaction. | SF-Hopkins Symptoms Checklist | Sociodemographic, health related and obstetrical history. | 6 -36 months |
| Cote-Corriveau G, 2022^20^ | Study of Hospital Clientele registry | Canada | Prospective cohort | 1381300 | Preterm birth defined as less than 37 weeks of gestation | Depression, bipolar disorder, stress and anxiety, personality disorder, intentional self-harm | ICD-9 and ICD-10 Codes | Age at first delivery, comorbidity, substance use disorders, socio economic status, rurality and time period. | 0-32 years |
| Singer L, 1999^21^ | Self-report interviews from mothers in Level III neonatal intensive care units | United States  1989 - 1992 | Prospective cohort | 329 | Preterm birth is defined as infants with VLBW admitted to NICU weighed less than 1500 g at birth and required oxygen. | Depression, Anxiety and OCD Behaviour | Brief Symptoms Inventory BSI Parenting Stress index PSI Impact on a family scale, FILE | Not reported | 3 years |

**Table S2: Characteristics of included studies reporting the association between pregnancy and birth complications and adverse long-term maternal mental health outcomes – Narrative report.**

| **Study, Year** | **Data Source** | **Country, Period** | **Study design** | **Sample Size** | **Definition of pregnancy/ birth complication** | **Defined mental outcomes.** | **Assessment method of Mental outcomes** | **Variables accounted for** | **Main Findings** |
| --- | --- | --- | --- | --- | --- | --- | --- | --- | --- |
| Engelhard I, 2002^22^ | Self-reported questionnaires, hospital records | Nether- land | Prospective cohort | 46 | *Preeclampsia* defined as requiring clinical management for preeclampsia for at least one week. | PTSD | DSM-IV, BDI, PDEQ, RIQ, WBSI, PTSD symptom scale PSS. | Age, marital status, educational status, gestational age at admission, features related to the delivery – birthweight, length of maternal hospitalisation | PTSD Criteria was met after PE by 28%, after PT by 28% and by control group by 0%. Results are statistically significant between the groups P=0.004. Adjusted estimates by length of time between birth and participation in the study was not significant between the PTSD cases. |
| Turton P, 2008^23^ | Self-reported questionnaires | United Kingdom  2003 -2006 | Case control | 103 | Stillbirth defined as spontaneous loss after 18 weeks gestation. | PTSD, Depression | DSM IV- (SCID) | Education, age, ethnic origin. | Study found that although case level PTSD had not persisted in the index group, women who had case level PTSD in the subsequent pregnancy continued, 7 years later, to regard their stillbirth as traumatic and to have significantly higher symptom levels than (p<.001) |
| Singer L, 2007^24^ | Self-report interviews from mothers in Level III neonatal intensive care units | United States  1989 - 1991 | Prospective cohort | 329 | Preterm birth is defined VLBW admitted to NICU less than 1500 g at birth and required oxygen. | Depression Anxiety and OCD Behaviour | BSI PSI Impact on a family scale, FILE | Not reported | At 8 years, there were no differences on the BSI summary score (F .61; P <.55) or any subscale. |
| Jacob L, 2019^25^ | Disease Analyser database (IQVIA) | Germany  2013 -2017 | Case  control | 57770 | Abortion defined according to ICD codes. | psychiatric disorders (depression, anxiety adjustment disorder, somatoform disorder) | ICD-10: F32, F33, F41, F43, F45 | Age, index year, observational time, and parity, benign breast disorders, inflammatory disorders of female pelvic organs. | Induced abortion (odds ratios [ORs] ranging from 1.75 to 2.01), Spontaneous abortion (ORs ranging from 2.16 to 2.60) were positively associated with the risk of psychiatric disorder. |
| Ahlund S, 2009^26^ | Not reported | United Kingdom 2004 - 2006 | Prospective cohort | 46 | Preterm birth is defined as gestational age <37 weeks. | PTSD | IES -R | Not reported | Compared with controls, mothers of VLBW infants recorded significantly higher levels of traumatic stress symptoms overall (median: controls, 0 (range 0–5) versus VLBW, 25 (range 2–82), P<0.001) |
| Roubinov D, 2022^27^ | Self- report Questionnaires | United States  2016 | Prospective cohort | 11320 | Preterm birth defined as gestational age <37 weeks. | Depressive symptoms | PROMS information systems, EPDS, ASR, BSI, CES-D, BDI, Kessler 6 Mental health Scale | Ethnicity, education, marital status, health insurance, substance use | Among mothers of preterm infants, a substantial proportion evidenced little or no depressive symptoms over time, suggestive of generally positive adjustment in the context of a significant life stressor. However, we observed a small (but clinically meaningful) group of mothers of preterm infants with initially mild depressive symptoms that increased to a severe level by the time offspring were 5 years of age. |
| Kersting A, 2004^28^ | SCID interview | Germany | Prospective cohort | 49 | Preterm birth defined as very low birth weight, < 32 gestation weeks. | Anxiety, depression, and traumatic experiences. | DSM-IV, SCID-I, IES, BDI, MADRS, STAI, HAMA | Not reported | The longitudinal section investigation of the mothers of the VLBW sample showed, surprisingly, that even 14 months after birth, the depressive and anxiety symptoms remained almost unchanged with respect to STAI-State. Preterm birth and depression: Mean SD 8.52 (8.65) control 4.00 (3.90)   t statistics 2.194 P-value 037, PTSD: IES total: Preterm birth 14.71 (13.38) control 3.86 (3.90) t statistics 3.598 P value: .002. Anxiety: Mean SD 41.48 (11.66)   control 34.27 (7.52 t-statistics 2.396, P value 0.22. |
| Henderson I, 2021^29^ | UK Millennium Cohort Study | United Kingdom | Prospective cohort | 15936 | Elective and emergency cesarean section defined according to ICD-10 codes. | Depression and anxiety | EPDS | Maternal age, ethnicity. Household income, education, ethnicity, parity, BMI, breastfed, epidural, induction, gestation at birth, admission to NICU. | Reported combined out-come of depression and anxiety. Women were at a greatest risk following emergency CS compared to women who had a vaginal birth at 3 years post-partum. The RR for ELCS in this group was 34% lower than expected multiplicatively, and 28% lower than expected additively. |
| Linden M, 2011^30^ | Self- report Questionnaires | Canada  2001 - 2004 | Prospective cohort | 150 | Preterm birth defined as gestational age <32 weeks, admitted to level III NICU. | Depression, Anxiety Parenting stress, Coping, Child behaviour, Child IQ | BDI-II, CBCL, STAI, WISC-IV, WCQ, PSI | Not reported | According to the BDI classification, two parents in the preterm group had severe depressive symptomatology, 3 had moderate and 10 were mild. One parent in the full-term group had a moderate level of depressive symptoms Preterm birth and depression: Depression measured in Median and range cases 4.0 (0-44) control 3.0 (0-25).3 Anxiety: measured in mean and SD cases 35.9 (9.25) control 34.2 (8.35). Group difference (p =0.012) |
| Adib-Rad H, 2019^31^ | Self-reported questionnaire | Iran  2015-2017 | Case  control | 355 | Recurrent Miscarriage (Not defined) | Somatization, OCD disorders, depression, anxiety. | SCL-90-R, IUS, GSI. | Age, level of education, BMI, occupation, residence/homeowner status, gravidity, time since last pregnancy. | In the control group, scores on all psychological distress subscales reduced significantly between 1-6 months to >12 months after birth (p=0.0001). However, in the RSA group, mental health problems remained stable even after 12 months since abortion. |
| Janssen H, 1996^32^ | Self-reported survey | Netherland & Belgium | Prospective cohort | 440 | Miscarriage defined as (early involuntary pregnancy loss <20 weeks) and (perinatal loss >20 weeks) . | Depression, anxiety, somatization, and OCD. | SCL -90 | Not reported | At 6 months the differences between the two groups were still substantial p< 0.0005). At 12 and 18 months, the difference between the two groups with regard to mental health symptoms no longer reached the 0.05 significance level. |
| Francisco M, 2014^33^ | Self-reported questionnaire | Brazil, 2011 | Case  control | 105 | Recurrent spontaneous abortion; women with history of two or more repeat spontaneous abortions | Depression and sexuality | BDI, FSFI | Not reported | The incidence of all levels of depression is approximately twice as high among patients in the RSA group when compared to those in the control group (test ƛ2^;^ p < 0.05). |

Global severity index (GSI). Impact of Event Scale (IES), Social Readjustment Rating Scale (RSS), Child Behaviour Checklist (CBCL), Beck Depression Inventory (BDI), State-Trait Anxiety Inventory (STAI), International Classification of Diseases (ICD), Patient-Reported Outcomes Measurements  (PROMS), Adults Self-Report (ASR) Depression Problems Syndrome Scale, Brief-Symptom Inventory (BSI),  Centre for Epidemiological Studies Depression Scale (CES-D), Ways of Coping Questionnaire (WCQ), , Parenting Stress Index (PSI), The Impact on Family Scale, the Family Inventory of Life Events and Changes (FILE), Post-traumatic Stress Disorder (PTSD), Structured Clinical Interview for DSM  (SCID), Peritraumatic Dissociative Experiences Questionnaire (PDEQ), Response to Intrusions Questionnaire (RIQ), the White Bear Suppression Inventory (WBSI) the PTSD Symptom Scale (PSS), Revised Symptom checklist-90 (SCL-90-R); Intolerance of Uncertainty Scale (IUS), Very low birthweight infants (VLBW), Montgomery Asberg Depression Scales (MADRS) State-Trait Anxiety Inventory (STAI), Hamilton Anxiety Scale (HAMA), Neonatal Intensive Care Unit (NICU), Recurrent Spontaneous Abortion (RSA), Obsessive Compulsive Disorders (OCD), Body Mass Index (BMI), Postpartum Depression (PPD), Elective Cesarean Section (ELCS), Emergency Cesarean Section (EMCS), Odds Ratio (OR), Diagnostic and Statistical Manual of Mental Disorders 4th Edition (DSM-IV), Standard Deviation (SD), Edinburgh Postnatal Depression Scale (EPDS), Intelligent Quota (IQ).  Perinatal Grief Scale (PGS). The Preeclampsia Risk Evaluation in FEMales (PREVFEM), International Society for the Study of Hypertension in Pregnancy (ISSHP), Fatigue Assessment Scale (FAS), Healthcare Cost and Utilisation Project (HCUP), severe intrapartum maternal morbidity (SMM), Delusions-Symptoms-States Inventory (DSSI), Mater-University of Queensland Study of Pregnancy (MUSP), Termination of pregnancy (TOP), Perinatal Risk Inventory (PRI), Environmental Influences on Child health Outcomes (ECHO), Socio-economic Status (SES), Victorian Infant Brain Studies (Vibes), General Health Questionnaire (GHQ), the Hospital Anxiety and Depression Scale (HADS), Post-traumatic Stress Disorder Checklist Civilian Version (PCL-S), The Self-rating Anxiety Scale (SAS), Life Stressor Checklist (LSC), The Abuse Assessment Screen (AAS), National Women’s Study PTSD Module (NWS-PTSD) version of DSM-IV, Composite International Diagnostic Interview (CIDI), Perinatal PTSD Questionnaire (PPQ), Recurrent Pregnancy Loss (RPL), Major Depression Index (MDI) self-rating depression scale, The Female Sexual Function Index (FSFI).

**Table S3: Newcastle Ottawa studies quality assessment of included studies.**

|  | | | | | | | | | | | |
| --- | --- | --- | --- | --- | --- | --- | --- | --- | --- | --- | --- |
| Studies | **Selection** | | | | **Comparability** | | **Outcome** | | | **NOS Score** | |
|  | Representativeness of the exposed Cohort | Selection of the non-exposed cohort | Ascertainment of exposure | Demonstration that outcome of interest was not present at the start of study. | Comparability of cohorts on the basis of the design and analysis | | Assessment of outcome | Was follow-up long enough for outcomes to occur | Adequacy of follow up of the cohort |  |  |
| Steinberg J, 2014^1^ | * | * | * | * | * | - | - | * | * | 7 |  |
| Kersting A, 2007^2^ | - | * | * | - | - | - | - | * | - | 3 |  |
| Mommersteeg P, 2016^3^ | - | * | * | * | * | - | - | * | * | 6 |  |
| Lewkowitz A, 2019^4^ | * | * | * | * | * | - | * | * | * | 8 |  |
| Abajobir A, 2017^5^ | * | * | * | - | - | * | - | * | * | 6 |  |
| Chen H, 2022^6^ | * | * | * | * | * | - | * | * | * | 8 |  |
| Yaari M, 2019^7^ | * | * | * | - | * | - | - | * | * | 6 |  |
| Yates R, 2021^8^ | * | * | - | * | - | * | - | * | * | 6 |  |
| Huang Z, 2011^9^ | * | * | * | * | * | - | - | * | * | 7 |  |
| Jacob L, 2017^10^ | * | * | * | * | * | - | * | * | * | 8 |  |
| Schmiege S, 2005^11^ | * | * | * | - | * | - | - | * | * | 6 |  |
| Gong X, 2012^12^ | * | * | * | * | * | - | - | * | * | 7 |  |
| Hamama L, 2010^13^ | * | * | * | * | - | * | - | * | * | 7 |  |
| Suttora C, 2014^14^ | - | * | - | - | - | - | - | * | * | 4 |  |
| Dingle K, 2008^15^ | * | - | * | - | * | - | - | * | - | 4 |  |
| Kolte A, 2015^16^ | - | - | * | * | - | * | - | * | * | 5 |  |
| Auger N, 2020^17^ | * | * | * | * | * | - | * | * | * | 8 |  |
| Hernandez - Martinez A, 2019^18^ | * | * | * | - | * | * | - | * | * | 7 |  |
| Gravensteen I, 2018^19^ | * | * | * | - | * | - | * | * | * | 7 |  |
| Cote-Corriveau G, 2022^20^ | * | * | * | * | * | - | * | * | * | 8 |  |
| Singer L, 1999^21^ | * | - | * | - | - | - | - | * | * | 4 |  |
| Janssen H, 1996^32^ | * | * | - | - | - | - | - | * | * | 5 |  |
| Engelhard I, 2002^22^ | - | * | * | - | - | - | - | * | * | 6 |  |
| Ahlund S, 2009^26^ | * | * | * | - | - | - | - | * | - | 4 |  |
| Roubinov D, 2022^27^ | * | * | * | * | - | - | - | * | * | 6 |  |
| Henderson I, 2021^29^ | * | * | - | * | * | - | - | * | * | 6 |  |
| Linden M, 2011^30^ | * | - | * | - | - | - | - | * | * | 4 |  |
| Singer L, 2007^24^ | * | - | * | * | - | - | - | * | * | 5 |  |
| Francisco M, 2014^33^ | - | - | * | * | - | - | * | * | * | 5 |  |
| Kersting A, 2004^28^ | - | * | - | - | - | - | - | * | - | 2 |  |
| **CASE-CONTROL STUDIES** | | | | | | | | | | | |
| Author, Year | **Selection** | | | | **Comparability** | | **Exposure** | | | **NOS Score** | |
|  | Is the case definition adequate? | Representativeness of the cases | Selection of Controls | Definition of Controls | Comparability of cases and controls on the basis of the design or analysis | | Ascertainment of exposure | Same method of ascertainment for cases and controls | Non -Response rate |  |  |
| Jacob L, 2019^25^ | * | * | * | * | * | - | * | * | * | 8 |  |
| Adib-Rad H, 2019^31^ | * | - | - | * | * | * | * | * | - | 6 |  |
| Turton P, 2008^23^ | * | * | - | - | * | - | * | * | - | 5 |  |
|  | | | | | | | | | | | |
|  | | | | | | | | | | | |

**References**

1. Steinberg JR, McCulloch CE, Adler NE. Abortion and mental health: findings from The National Comorbidity Survey-Replication. *Obstetrics and gynecology*. 2014;123(2 Pt 1):263-270. doi:10.1097/AOG.0000000000000092

2. Kersting A, Kroker K, Steinhard J, et al. Complicated grief after traumatic loss: a 14-month follow up study. *Eur Arch Psychiatry Clin Neurosci*. 2007;257(8):437-443. doi:10.1007/S00406-007-0743-1

3. Mommersteeg PMC, Drost JT, Ottervanger JP, Maas AHEM. Long-term follow-up of psychosocial distress after early onset preeclampsia: the Preeclampsia Risk EValuation in FEMales cohort study. *http://dx.doi.org/103109/0167482X20161168396*. 2016;37(3):101-109. doi:10.3109/0167482X.2016.1168396

4. Lewkowitz AK, Rosenbloom JI, Keller M, et al. 121: Is stillbirth associated with increased risk of severe psychiatric illness within the subsequent year? *Am J Obstet Gynecol*. 2019;220(1):S96. doi:10.1016/j.ajog.2018.11.142

5. Abajobir AA, Alati R, Kisely S, Najman JM. Are Past Adverse Pregnancy Outcomes Associated with Maternal Anxiety and Depressive Symptoms in a Sample of Currently Pregnant Women? *Ethiop J Health Sci*. 2017;27(4):351-362. doi:10.4314/EJHS.V27I4.6

6. Chen HH, Lai JCY, Hwang SJ, Huang N, Chou YJ, Chien LY. Understanding the relationship between cesarean birth and stress, anxiety, and depression after childbirth: A nationwide cohort study. *Birth*. 2017;44(4):369-376. doi:10.1111/BIRT.12295

7. Yaari M, Treyvaud K, Lee KJ, Doyle LW, Anderson PJ. Preterm Birth and Maternal Mental Health: Longitudinal Trajectories and Predictors. *J Pediatr Psychol*. 2019;44(6):736-747. doi:10.1093/JPEPSY/JSZ019

8. Yates R, Anderson PJ, Lee KJ, et al. Maternal Mental Health Disorders Following Very Preterm Birth at 5 Years Post-Birth. *J Pediatr Psychol*. 2022;47(3):327-336. doi:10.1093/JPEPSY/JSAB101

9. Huang Z, Hao J, Su P, et al. The Impact of Prior Abortion on Anxiety and Depression Symptoms During a Subsequent Pregnancy: Data From a Population-Based Cohort Study in China. *http://dx.doi.org/105455/bcp20111102040509*. 2016;22(1):51-58. doi:10.5455/BCP.20111102040509

10. Jacob L, Polly I, Kalder M, Kostev K. Prevalence of depression, anxiety, and adjustment disorders in women with spontaneous abortion in Germany - A retrospective cohort study. *Psychiatry Res*. 2017;258:382-386. doi:10.1016/J.PSYCHRES.2017.08.064

11. Schmiege S, Russo NF. Depression and unwanted first pregnancy: longitudinal cohort study. *BMJ : British Medical Journal*. 2005;331(7528):1303. doi:10.1136/BMJ.38623.532384.55

12. Gong X, Hao J, Tao F, Zhang J, Wang H, Xu R. Pregnancy loss and anxiety and depression during subsequent pregnancies: data from the C-ABC study. *Eur J Obstet Gynecol Reprod Biol*. 2013;166(1):30-36. doi:10.1016/J.EJOGRB.2012.09.024

13. Hamama L, Rauch SAM, Sperlich M, Defever E, Seng JS. Previous experience of spontaneous or elective abortion and risk for posttraumatic stress and depression during subsequent pregnancy. *Depress Anxiety*. 2010;27(8):699-707. doi:10.1002/DA.20714

14. Suttora C, Spinelli M, Monzani D. From prematurity to parenting stress: The mediating role of perinatal post-traumatic stress disorder. *http://dx.doi.org/101080/174056292013859574*. 2014;11(4):478-493. doi:10.1080/17405629.2013.859574

15. Dingle K, Alati R, Clavarino A, Najman JM, Williams GM. Pregnancy loss and psychiatric disorders in young women: an Australian birth cohort study. *The British Journal of Psychiatry*. 2008;193(6):455-460. doi:10.1192/BJP.BP.108.055079

16. Kolte AM, Olsen LR, Mikkelsen EM, Christiansen OB, Nielsen HS. Depression and emotional stress is highly prevalent among women with recurrent pregnancy loss. *Human Reproduction*. 2015;30(4):777-782. doi:10.1093/HUMREP/DEV014

17. Auger N, Low N, Paradis G, Ayoub A, Fraser WD. Preeclampsia and the longitudinal risk of hospitalization for depression at 28 years. *Soc Psychiatry Psychiatr Epidemiol*. 2021;56(3):429-436. doi:10.1007/S00127-020-01920-X/METRICS

18. Hernández-Martínez A, Rodríguez-Almagro J, Molina-Alarcón M, Infante-Torres N, Rubio-Álvarez A, Martínez-Galiano JM. Perinatal factors related to post-traumatic stress disorder symptoms 1-5 years following birth. *Women Birth*. 2020;33(2):e129-e135. doi:10.1016/J.WOMBI.2019.03.008

19. Gravensteen IK, Jacobsen EM, Sandset PM, et al. Anxiety, depression and relationship satisfaction in the pregnancy following stillbirth and after the birth of a live-born baby: A prospective study. *BMC Pregnancy Childbirth*. 2018;18(1):1-10. doi:10.1186/S12884-018-1666-8/FIGURES/2

20. Côté-Corriveau G, Paradis G, Luu TM, Ayoub A, Bilodeau-Bertrand M, Auger N. Longitudinal risk of maternal hospitalization for mental illness following preterm birth. *BMC Med*. 2022;20(1):1-8. doi:10.1186/S12916-022-02659-9/FIGURES/3

21. Singer LT, Salvator A, Guo S, Collin M, Lilien L, Baley J. Maternal psychological distress and parenting stress after the birth of a very low-birth-weight infant. *JAMA*. 1999;281(9):799-805. doi:10.1001/JAMA.281.9.799

22. Engelhard IM, Van Rij M, Boullart I, et al. Posttraumatic stress disorder after pre-eclampsia: An exploratory study. *Gen Hosp Psychiatry*. 2002;24(4):260-264. doi:10.1016/S0163-8343(02)00189-5

23. Turton P, Evans C, Hughes P. Long-term psychosocial sequelae of stillbirth: Phase II of a nested case-control cohort study. *Arch Womens Ment Health*. 2009;12(1):35-41. doi:10.1007/S00737-008-0040-7/METRICS

24. Singer LT, Fulton S, Kirchner HL, et al. Parenting very low birth weight children at school age: maternal stress and coping. *J Pediatr*. 2007;151(5):463-469. doi:10.1016/J.JPEDS.2007.04.012

25. Jacob L, Gerhard C, Kostev K, Kalder M. Association between induced abortion, spontaneous abortion, and infertility respectively and the risk of psychiatric disorders in 57,770 women followed in gynecological practices in Germany. *J Affect Disord*. 2019;251:107-113. doi:10.1016/J.JAD.2019.03.060

26. Åhlund S, Clarke P, Hill J, Ks Thalange N. Post-traumatic stress symptoms in mothers of very low birth weight infants 2-3 years post-partum. *Arch Womens Ment Health*. 2009;12(4):261-264. doi:10.1007/S00737-009-0067-4

27. Roubinov D, Musci RJ, Hipwell AE, et al. Trajectories of depressive symptoms among mothers of preterm and full-term infants in a national sample. *Arch Womens Ment Health*. 2022;25(4):807-817. doi:10.1007/S00737-022-01245-5

28. Kersting A, Dorsch M, Wesselmann U, et al. Maternal posttraumatic stress response after the birth of a very low-birth-weight infant. *J Psychosom Res*. 2004;57(5):473-476. doi:10.1016/J.JPSYCHORES.2004.03.011

29. Henderson I, Quenby S. The association between caesarean and postnatal psychological distress: Effect modification by mental health history. *Paediatr Perinat Epidemiol*. 2021;35(6):635-644. doi:10.1111/PPE.12791

30. Linden MA, Cepeda IL, Synnes A, Grunau RE. Stress in parents of children born very preterm is predicted by child externalising behaviour and parent coping at age 7 years. *Arch Dis Child*. 2015;100(6):554-558. doi:10.1136/ARCHDISCHILD-2014-307390

31. Adib-Rad H, Basirat Z, Faramarzi M, Mostafazadeh A, Bijani A. Psychological distress in women with recurrent spontaneous abortion: A case-control study. *Turk J Obstet Gynecol*. 2019;16(3):151. doi:10.4274/TJOD.GALENOS.2019.88899

32. Janssen HJEM, Cuisinier MCJ, Hoogduin KAL, De Graauw KPHM. Controlled prospective study on the mental health of women following pregnancy loss. *Am J Psychiatry*. 1996;153(2):226-230. doi:10.1176/AJP.153.2.226

33. Francisco MDFR, Mattar R, Bortoletti FF, Nakamura MU. [Sexuality and depression among pregnant women with recurrent spontaneous abortion]. *Rev Bras Ginecol Obstet*. 2014;36(4):152-156. doi:10.1590/S0100-720320140050.0004
